# The potential roles of aquaporin 4 in malignant gliomas

**DOI:** 10.18632/oncotarget.16017

**Published:** 2017-03-08

**Authors:** Yu-Long Lan, Xun Wang, Jia-Cheng Lou, Xiao-Chi Ma, Bo Zhang

**Affiliations:** ^1^ Department of Neurosurgery, The Second Affiliated Hospital of Dalian Medical University, Dalian, China; ^2^ Department of Pharmacy, Dalian Medical University, Dalian, China; ^3^ Department of Physiology, Dalian Medical University, Dalian, China; ^4^ Department of Neurosurgery, The Third Peoples Hospital of Dalian, Non-Directly Affiliated Hospital of Dalian Medical University, Dalian, China

**Keywords:** AQP4, orthogonal arrays of particles, glioma, expression, regulation

## Abstract

Aquaporin 4 (AQP4) is the major water channel expressed in the central nervous system and is primarily expressed in astrocytes. Recently, accumulated evidence has pointed to AQP4 as a key molecule that could play a critical role in glioma development. Discoveries of the role of AQP4 in cell migration suggest that AQP4 could be a significant factor regarding glioma malignancies. However, the AQP4 expression levels in glioma have not been fully elucidated; furthermore, the correlation of AQP4 expression with glioma malignancy remains controversial. Here, we review the expression pattern and predictive significance of AQP4 in malignant glioma. The molecular mechanism of AQP4 as it pertains to the migration and invasion of human glioma cells has been summarized. In addition, the important roles of AQP4 in combating drug resistance as well as potential pharmacological blockers of AQP4 have been systematically discussed. More research should be conducted to elucidate the potential roles of AQP4 in malignant glioma for identifying the tumor type, progression stages and optimal treatment strategies. The observed experimental results strongly emphasize the importance of this topic for future investigations.

## INTRODUCTION

To date, more than 13 water channel proteins (AQP1-13) have been identified in multiple mammalian species and appear to possess various regulatory functions in a wide variety of diseases, including brain edema [1–3], hepatoencephalopathy [4], seizures [5–6] and brain tumors [7]. AQP1, AQP4 and AQP9 have been clearly identified as brain-specific; among them, AQP4 is the most important family member that has been well described as participating in brain edema and other various brain diseases [8]. Intriguingly, a surprising link between AQPs and cancer as emerged in recent years. AQPs appear to play a key role in several tumor-related processes, including tumor edema, tumor cell migration, tumor proliferation and angiogenesis [9]; however, the mechanisms remain unclear.

Glioma is a frequently occurring malignant tumor in the central nervous system (CNS). Glioblastoma multiforme (GBM) is the most common and malignant type of brain tumors in adults. Most patients develop clinical symptoms over a short time span (approximately 3 months) and often die within 8-18 months of diagnosis [10]. Fewer than half the patients diagnosed with GBM live for longer than 6 months, and the 2-year survival rate is only 3% [11]. The global burden of malignant glioma is expected to rise, and its diffuse and invasive growth combined with its high frequency of recurrence are associated with a poor prognosis [12–14]. Due to the poor prognosis of these patients, new therapeutic approaches are urgently required. Current evidence has indicated that compared to low-grade tumors or normal brain tissue, high-grade tumors have up-regulated expression of AQP4, which possibly contributes to cerebral edema [15, 16]. Other authors have associated AQP4 with the regulation of human glioma cell migration and invasion [17, 18]. In addition, various studies have confirmed the increased expression of AQP4 in GBM, and the effects of down-regulated AQP4 in inducing glioblastoma cell apoptosis [19] have also been reported. All these data suggest the involvement of AQP4 in malignant brain tumors and indicated that AQP4 could serve as a potential target for therapy of glioma.

**Figure 1:**
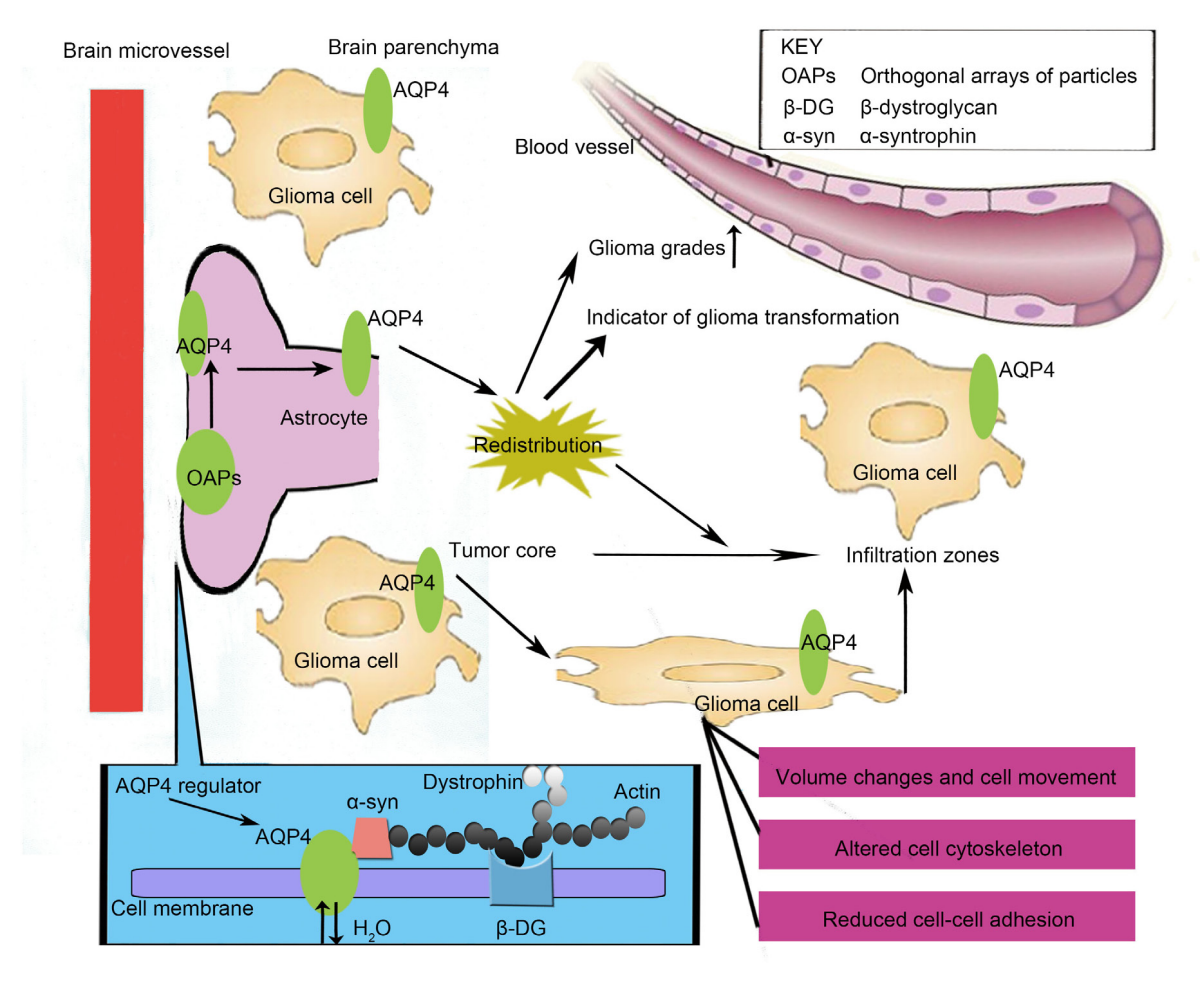
The new potential roles of AQP4 in glioma Under tumor conditions, AQP4 is dissociated from the orthogonal arrays of particles (OAPs) and is redistributed across the entire surface of the glioma cell. AQP4 expression levels could significantly correlate to the tumor grade, and it is generally accepted that AQP4 expression levels increase with higher glioma grades. More importantly, the redistribution of AQP4 and OAPs could be one of the earliest indicators of glioma transformation. However, the facilitating role of AQP4 in the infiltration and invasion of malignant cells in glioblastoma requires additional research. First, the localization of AQP4 to the leading edge of migrating tumor cells could provide evidence that water release is required to achieve any volume change during the process of cell migration, with AQP4 at the leading edge of the invading cell possibly serving this role. More importantly, AQP4 might be involved in cytoskeleton organization by affecting the actin cytoskeleton, β-dystroglycan (β-DG), α-syntrophin, dystrophin and/or utrophin. In addition, AQP4 may impact the control and maintenance of normal intercellular adhesion, which could be an essential step in the progression from a localized malignancy to metastatic disease, by interacting with adhesion-associated proteins.

Intriguingly, there are some discrepancies regarding the role of AQP4 in malignant gliomas. Using a pharmacological inhibitor and small-interfering RNA, Ding et al. [17] demonstrated that knocking down AQP4 expression could result in specific and massively impaired invasion and migration glioblastoma cells. The authors also confirmed that AQP4 is involved in the control of glioblastoma cell invasion and migration and could be a potential therapeutic target to combat glioblastoma cell infiltration. Aside from water transport, AQP4 has been shown to contribute to the regulation, invasion and migration of gliomas [17, 18]. Previously, McCoy et al. [20] found that by stably reintroducing either AQP1 or AQP4 into glioma cell lines, AQP4 could actually reduce the migratory ability of the cell, which is inconsistent with the results of the study performed by Ding et al. [17, 18]. However, this may be due to their use of the M23 splice variant of AQP4, which has been shown to increase cell adhesiveness [21] rather than cell migration and invasion. Another study by McCoy's group revealed that glioma cells expressing AQP4 showed significantly reduced invasion abilities compared to tumors expressing AQP1 as determined by quantitative stereology; these results were consistent with the viewpoint regarding the differential roles for AQP1 and AQP4 during tumor progression [22]. These findings could serve as a basis in unraveling the controversy and mystery of AQP4 function in glioma. Accumulated evidence has pointed to AQP4 as a key molecule that plays an important role in glioma development [23]; here, we review the potential roles of AQP4 in malignant glioma (Figure 1)

## THE EXPRESSION PATTERN OF AQP4 IN HUMAN GLIOMA TISSUE

The arrangement of AQP4 is morphologically spectacular, as it forms orthogonal arrays of particles (OAPs) that can be observed in freeze-fracture replicas. In brain tumors, these OAPs are redistributed to membrane domains apart from end-feet areas and are redistributed across the entire surface of the cell [24]. Wolburg et al. [24] reviewed the disruption of the blood-brain barrier (BBB) in human glioblastoma. It is now accepted that astrocytes, which were frequently shown to express AQP4 and OAPs, could strictly control the maintenance of the BBB [25, 26] by encompassing the vessels by sending an end-foot toward the perivascular basal lamina [27]. During brain development, an AQP4/OAP-related polarity (i.e., the ratio of OAP densities in end-foot versus non-end-foot membranes) is required for astrocytes, and this has been demonstrated to be important for the induction and/or maintenance of the BBB [28, 29]. Thus, it is assumed that AQP4 in astrocytes may significantly contribute to the aberrant function of the BBB in human glioblastoma. Using freeze-fracture replicas and immunogold staining against AQP4, Wolburg et al. [24] attempted to observe the OAP expression profile in normal and glioblastoma tissues from humans. They found that OAPs localize to the parenchymal membrane domains (which are normally absent in glioblastoma), whereas the high density observed in the normal astroglial end-feet membranes disappear in glioma cell membranes, including those in contact with the basal lamina. Noell et al. [30] demonstrated that this disrupted localization could be caused by the degradation of the proteoglycan agrin; intriguingly, the presence of agrin is indispensable for the formation of OAPs. Loss of agrin may lead to AQP4 redistribution and compromised directionality of water transport out of the cell, which can destroy the BBB and cause brain edema [31]. The authors also confirmed the important role of the dystrophin-dystroglycan complex (DDC) for the adequate expression of AQP4 at the BBB [32], and these results were consistent with those from a previous study conducted by Warth et al. [33] who suggested that AQP4 is tightly associated with the DDC and that agrin is indispensable for the polarized distribution of AQP4 in astrocytes.

Wolburg et al. [24] also confirmed that in tumors, the entire glioma cell shows strong AQP4 staining; however, the overall density of the OAPs is not nearly as high as that in the normal end-foot membrane, even near vessels. Thus, it could be assumed that in glioma cells, AQP4 might also exert activities independent of OAPs. It has been reported that AQP4 could dissociate from OAPs and redistribute across the entire surface of tumor cells [30, 31, 32, 33]. Currently, the functional differences between AQP4 within OAPs and standalone AQP4 still require further research. Analysis of the composition of freeze-fractured membranes from cells transfected with the M23, M1 or a mixture of both M1 and M23 isoforms of AQP4 has been conducted by Furman et al. [34]. Interestingly, only transfection with both isoforms resulted in the formation of OAPs. Thus, specific up-regulation of the M1 isoform in glioma might explain the interesting phenomenon regarding up-regulated AQP4 in conjunction with down-regulated OAPs.

However, a study by Noell et al. [35] presented different results. In their experiments with human and glioma tissue, they did not observe OAPs despite the presence of both AQP4-M1 and AQP4-M23. This was completely unexpected because expression of both the M1 and M23 isoforms of AQP4 should result in the formation of OAPs. This raises the question of whether the informative value of transfected cell lines could translate to *in vivo* situations. More efforts should be directed toward clarifying how *in vitro* culture conditions affect AQP4 expression. Previously, McCoy et al. [20] confirmed that all glioma patient biopsies express AQP1 and AQP4, with some samples showing expression of AQP5; however, when isolated and grown as cell lines, no AQP proteins could be detected except for AQP1 in a small subset of cell lines. Noell et al. [35] also confirmed the loss of AQP4 in primary human glioblastoma cell cultures after a few passages. Interestingly, the authors also indicated for the first time that AQP4-negative glioma cells implanted in the animal brain or flank could specifically express AQP4 in intracerebral gliomas but neither extracranial nor flank gliomas had detectable AQP4 expression. Thus, certain intracerebral factors may be necessary for AQP4 expression. It is currently unclear how the brain microenvironment affects AQP4 expression, and more research should be conducted on this topic.

## AQP4 MAY BE A MARKER FOR THE DIAGNOSIS AND PROGRESSION OF HUMAN MALIGNANT GLIOMA

Recent studies have called special attention to AQPs as potential diagnostic and therapeutic biomarkers [36]. AQP4, which is the water channel with by far the highest water flux capacity in the brain, has been found to be strongly up-regulated and redistributed across the entire surface of all glial tumor cells [15, 16, 24, 30, 33, 37] as mentioned above. The redistribution and the displaced insertion of AQP4 molecules, which are a consequence of severe alterations of the microenvironment, are part of a set of stereotypical responses leading to the most serious clinical signs of glioblastoma—brain edema [38]. Tumor-associated edema significantly contributes to the mass effects of glioma and neurological deterioration. In recent decades, the pathophysiological mechanisms leading to the development of edema have been characterized in a stepwise manner [39]. It is generally accepted that tumor-related edema is considered vasogenic, i.e., disturbed BBB function resulting in increased vascular permeability. The altered vascular architecture in brain tumors results in the loss of barrier function and allows plasma fluid and proteins to leak into the surrounding tissue [40]. In addition, other factors that could cause BBB dysfunction might actually be the source of tumor-associated edema. For example, tumor cells typically produce various cytokines that act on endothelial cells located within or around the tumor microenvironment, and the most important cytokine secreted by various brain tumors is VEGF [41]. Under the influence of VEGF, the permeability of the endothelium is increased, resulting in the disturbance of the BBB and tumor-associated edema [42, 43]. Interestingly, it was reported that after cerebral hypoxia and BBB disruption, VEGF co-localizes with AQP4 on astrocyte processes [44]. Moreover, an intracerebral injection of VEGF profoundly up-regulated AQP4 mRNA and protein levels in the perivascular space and glia limitans externa [45]. Based on evidence that VEGF is closely associated with AQP4 and that both molecules are essential to brain edema, it is speculated that the effect of VEGF on brain edema may result from its regulation of AQP4 expression. Furthermore, Chu et al. [46] demonstrated that VEGF may regulate AQP4 expression by activating MAPK pathways. VEGF can induce angiogenesis, and AQP4 and VEGF likely act in concert during the process of tumor-associated edema formation [47]. Thus, AQP4 expression could also affect angiogenesis, which is directly related to the incidence of edema. All these pathways could cause brain edema in glioma, and improved understanding of the molecular determinants underlying edema formation is a prerequisite for developing novel therapeutic agents with anti-edema and anti-tumor activity.

In addition, a correlation between increased BBB permeability and elevated AQP4 levels has been observed [15], and AQP4 up-regulation is also associated with brain edema formation in malignant gliomas [48]. Therefore, AQP4 could be regarded as a protective factor for the reduction of cerebral fluid accumulation in human gliomas, and a correlation between the degree of peritumoral edema and the expression level of AQP4 in peritumor could exist [23]. It is assumed that AQP4 expression levels could correlate to the tumor grade, with the generally accepted viewpoint that AQP4 expression increases with higher glioma grades [23]. A recent study by Zhao et al. [49] found weak positivity of AQP4 expression surrounding the capillary vessel in low-grade human glioma tissues, whereas in higher grade samples, the distribution of AQP4 was not confined to membranes contacting the basal laminae, which is involved in the construction of the BBB. This further confirms previous reports regarding the redistribution of AQP4 with the increase of the glioma grade. Thus, AQP4 and its redistribution might also be grade-dependent. One publication suggested that AQP4 could be used as a diagnostic marker to distinguish oligodendroglial and astrocytic tumors [50]. Similarly, another report [48] implied that AQP4 might be used to distinguish different WHO grades of astroglial tumors.

Warth et al. [48] investigated 189 primary gliomas of WHO grade I-IV along with 31 recurring glioblastoma tumors to obtain a more in-depth understanding regarding the role of AQP4 in human brain tumor edema and, more importantly, to explore whether a direct association between AQP4 expression levels and mortality could be found. Unfortunately, similar overall survival was found among patients with low, medium, and high AQP4 expression within each WHO group. Although the authors did not see an association of AQP4 expression and survival time among any of the WHO groups, they demonstrated that AQP4 up-regulation and redistribution could serve as a tumor progression marker in WHO grade II-IV astrocytomas, whereas unexpectedly high AQP4 expression levels were observed in WHO grade I pilocytic astrocytomas [48].

The authors additionally conducted an analysis regarding the comparison between AQP4 expression in glioblastoma centers and corresponding infiltration zones to identify whether different AQP4 levels between the tumor core and the infiltration zones are correlated to patient survival [48]. Compared to the results from a study performed by Mou et al. [23], who reported that AQP4 expression was higher in the peritumor than that in the tumor, Warth and colleagues unexpectedly observed significantly higher AQP4 expression levels within the tumor center than that observed in the peritumor, suggesting that the alterations in the AQP4 expression pattern are specific to neoplastic cells and are therefore irrelevant to counteracting the ability to prevent edema formation in tumor infiltration zones. Despite this controversy, comparisons between the tumor center and perifocal infiltration zone regarding AQP4 expression levels might be the key to clarifying the role of the AQP4 expression profile in predicting tumor progression. The redistribution of AQP4 and OAPs could be observed in the infiltration zone around the tumor proper in which the neurovascular unit appeared normal and histologically unaltered regarding the morphology [38]. This suggests that the redistribution of water channels is one of the earliest indicators of glioma transformation. Furthermore, compared to primary tumors, recurrent glioblastoma tumors did not appear to exhibit significant differences in their AQP4 expression levels [48]. However, these findings should be interpreted with caution given the small samples, but this is a very interesting topic that warrants more exploration.

## AQP4 COULD REGULATE THE MIGRATION AND INVASION OF HUMAN GLIOMA CELLS

It has been postulated that the AQP4 expression levels are relevant to the migration of astrocytes [51], indicating an enabling role of AQP4 in the infiltration of malignant glioblastoma cells. This may indicate that inhibiting AQP4 expression can limit infiltration. McCoy et al. [22] hypothesized that the invasive migration of glioma cells requires changes in cell volume; specifically, cell shrinkage to allow invasion through narrow extracellular spaces in the brain [52]. In addition, McCoy et al. [20] previously showed AQP4 localization at the leading edge of migrating tumor cells; thus, we could assume that water release is required to achieve any volume change in the process of cell migration, with AQP4 expression at the leading edge of an invading cell possibly serving this role [22]. Furthermore, the authors indicated that AQP4 localizes to the leading edge of the invadopodia of migrating cells and co-localizes with CLC2 and KCC1, which could be the mechanism by which Cl- and K^+^ are released during cell migration [53]. This provides further support for the role of KCl-water extrusion as an enhancer of cell invasion. To date, several studies have demonstrated AQP4-mediated migration [51, 54, 55, 56], but the mechanisms of the extent of AQP4 involvement in malignant glioma still requires more research.

The invasion of malignant glioma is a complex process most likely controlled by different genes and signaling pathways at multiple steps: reduced adhesion between tumor cells and other surrounding cells, increased tumor cell mobility, and degradation of the extracellular matrix [57]. Currently, the effect of AQP4 in promoting glioma cell migration and invasion can be summarized by the results of several key studies. First, the formation and retraction of cell membrane protrusions at the leading edge of glioma cells are essential for the migration and invasion of gliomas [58]. Interestingly, AQP4 polarizes to the lamellipodia and induces an increase in the number and/or size of lamellipodia in migrating cells, where there is rapid transmembrane water movement [55, 59]. This is consistent with reports indicating that ion channels and transporters could exert important effects on cell migration by polarizing the leading edge of migrating cells. The movement of these ions might contribute to an osmotic gradient that drives water influx during cell movement [60]. Second, AQP4 might be involved in cytoskeletal organization. A recent study has indicated that AQP4 deficiency in rat and human cells could be associated with actin depolymerization well as with a dramatic change of morphology: in astrocytes from AQP4 knockout mice, the F-actin cytoskeleton rearrangement in the cortical layer of the brain was completely replaced by fibers with a star-like organization [61]. Moreover, AQP4 could interact with α-syntrophin, which is a member of the DDC. This complex includes dystrophin and utrophin, linker molecules between the actin cytoskeleton and β-dystroglycan (β-DG) [33]. These findings strongly indicate the involvement of AQP4 protein in altering the cellular cytoskeleton in a manner that could play the decisive role in cell migration. Third, cell-cell adhesion determines cell polarity and could be generally reduced in human cancers. It is known that reduced intercellular adhesion promotes cell invasion, which is an essential step in the progression from localized primary malignancy to metastatic disease. The maintenance and control of normal intercellular adhesion is often regulated by the cadherin-catenin cell adhesion complex [18]; specifically, connexin 43 could be critical in calcium-dependent intercellular adhesion events [62] and is commonly overexpressed in certain types of cancer. For example, β-catenin binds the cytoplasmic domain of the cadherin adhesion receptors as well as with actin to bridge the extracellular adhesive activity of cadherins with the underlying actin cytoskeleton [63]. Dysregulation of these proteins can result in the loss of cell-cell adhesions. Nicchia et al. reported the possible novel roles of AQP4 and its functional relationship with connexin 43 regarding the cytoskeleton in astrocytes from AQP4 knockout mouse; these data could indicate a functional relationship between water channels and gap junctions in brain [61]. Thus, AQP4 could likely regulate glioma adhesion via adhesion-associated proteins such as connexin 43.

As mentioned above, aside from water transport, other potential roles of AQP4 in the regulation, invasion and migration of gliomas have been slowly elucidated. These properties of AQP4 appear unrelated to the canonical and well-established role of AQP4 in edema formation and absorption, suggesting this protein as novel therapeutic target for treating brain tumors [2, 64].

## AQP4 MIGHT BE A SIGNIFICANT CONTRIBUTOR TO BBB INTEGRITY AND DRUG RESISTANCE

There are various controversies that should be discussed. Because disrupting the BBB is an important pathophysiological change during the progression of glioblastoma and could play a critical role in the development of drug resistance in the treatment process, the roles of AQP4 in the BBB is an especially contentious issue. Zhou et al. [65] found that altered ultrastructures of brain microvessels, including open tight junctions and swollen perivascular astrocytic end-feet, were frequently observed in AQP4 knockout mice. The authors suggested that AQP4 is essential for maintaining BBB integrity, and reduced or absent expression could cause severe alterations in the function of endothelial and astroglial cells in the BBB. Because the BBB plays an important role in drug resistance during glioma chemotherapy, combating the resistance by targeting the BBB has become a heated topic that warrants more research. AQP4 might be an appropriate focal point; however, although a study conducted by Zhou et al. clearly showed that AQP4 deletion impaired tight junctions and BBB permeability, AQP4 knockout mice did not exhibit gross anatomic abnormalities. Disruption of the BBB as described in their Nanjing AQP4 knockout mice is severe [65], and their paper indicated that the BBB in AQP4 knockout mice is highly permeable to large molecules such as horseradish peroxidase (HRP). In contrast, when Saadoun et al. [66] investigated BBB permeability in the same Nanjing AQP4 knockout mice using the Evans Blue dye technique, they actually found no difference in BBB permeability between the WT and AQP4 knockout mice, which suggests that technical artifacts may have influenced the data reported by Zhou et al. and that compared to WT mice, AQP4 knockout mice present an expanded extracellular space as the only established brain phenotype. In contrast to the study by Zhou et al. [65], Saadoun et al. [66] also indicated that AQP4 deletion in mice might not alter brain characteristics, including BBB permeability. However, they used the San Francisco AQP4 knockout mice rather than the Nanjing AQP4 knockoutmice. Furthermore, Eilert-Olsen et al. [67] also demonstrated that deletion of AQP4 did not alter the ultrastructures of capillary endothelial cells, the expression of tight junction proteins or the vascular permeability to either HRP or Evans blue albumin dye. The authors concluded that AQP4 deletion could reduce the expression of perivascular glial scaffolding proteins without affecting the endothelial barrier. Their results were obtained in C57BL/6J mice and agree with those obtained in CD1 mice by Saadoun et al. but contrast those of Zhou et al., who also used the CD1 mice. Notably, a study by Feng et al. [3] failed to replicate the BBB dysfunction in the same mouse strain used by Zhou et al. More research should be directed toward clarifying this controversy, as AQP4-related functional changes of the BBB might be a potential new target for combating drug-resistant brain tumors and other CNS diseases that involve BBB dysfunction.

In addition, AQP4 might impact drug resistance through the ion transporter Na^+^/K^+^-ATPase. It is known that decreasing the migratory ability of glioma cells is a potential means of overcoming resistance, and involvement of the Na^+^/K^+^-ATPase in the migration and proliferation of glioma cells has been previously confirmed [68]. It has also been emphasized that a decrease in Na^+^/K^+^-ATPase activity could be used to combat apoptosis-resistant malignant gliomas. Furthermore, specific inhibition of Na^+^/K^+^-ATPase activity could disorganize the actin cytoskeleton (thus reducing motility and proliferation) and induce autophagy in malignant glioma models [69]. A study conducted by Thrane et al. [70] provided the first line of evidence that AQP4 could impact the oxygenation of brain tissue. The authors demonstrated that K^+^ uptake is suppressed in AQP4 knockout mice because of decreased oxygen delivery to tissue located farthest away from a vascular oxygen source. This finding could expedite research regarding Na^+^/K^+^-ATPase inhibition via AQP-4 mediated regulation of an ion imbalance. Thus, inhibiting oxygen delivery in AQP4-deficient cells may represent another important factor in regulating Na^+^/K^+^-ATPase activity and ultimately drug resistance.

## NOVEL PHARMACOLOGICAL INHIBITORS OF AQP4 MIGHT CRITICALLY AFFECT THE TREATMENT OF GLIOBLASTOMA

Collectively, the abovementioned findings suggest that the cancer-enhancing activity of AQP4 does more than simply create pathways for water flow and that additional distinguishing features could also be important. The role of AQP4 in human cancer has emerged as an area of intense research interest. Aquaporin inhibitors may therefore be a novel class of anti-tumor agents. Unfortunately, no such inhibitors are available to date, and attempts to produce small molecule inhibitors targeting aquaporins have been largely unsuccessful [9]. Specifically, there is no widely accepted specific AQP4 inhibitor. Screening for specific inhibitors of AQP4 may illuminate the design of novel mechanism-based therapies for glioma and further elucidate this important biological aspect of glioma progression. Thus, we will review potential AQP4 inhibitors that could serve as a basis for increased research interest and warrant more detailed exploration.

2-(Nicotinamide)-1,3,4-thiadiazole (TGN-020) is a low molecular weight compound that has recently been identified as an inhibitor of AQP4 [71]. The authors showed that TGN-020 could reduce ischemic cerebral edema *in vivo* [72]. However, there is no information regarding the influence of putative inhibitors on the M1 and M23 isoforms of AQP4, and no information regarding the association between inhibition of water transport and inhibition of tumor cell migration exists [73].

Propofol (2,6-dilsopropylphenol), an IV anesthetic commonly used in clinical practice, has been previously proven to influence neuronal apoptosis [74] and reduce AQP expression and brain edema in animal models [75]. Yang et al. [76] found that propofol could inhibit AQP4 expression. Currently, there are few studies that clinically connected to the action of propofol and AQP activity, and the mechanism of propofol on inhibiting AQP4 remains unclear.

An article published by Gunnarson et al. [77] has shown that protein kinase C (PKC) may be one of the potential kinases that targets AQP4 and influences the water permeability. Kleindienst et al. [78] also reported that PKC activators could inhibit ischemia-induced elevation of AQP4 expression in an animal model. PKC-mediated phosphorylation could be the target for many of the general anesthetic effects observed in the CNS, including propofol as mentioned above [79]. However, PKC activators might be too ubiquitous for use in humans.

Tetraethylammonium (TEA) has been indicated to inhibit water permeability of AQP4 in transfected oocytes [80] and has also been suggested to decrease water permeability in primary mouse astrocyte cultures [81]. Unfortunately, TEA is known to affect multiple ion transporters and has been shown to considerably change the electrochemical properties of cellular membranes [82].

Arylsulfonamides are a class of compounds that have shown promise for the development of pharmacological agents targeting AQPs [83]. Sulfonamides, including the carbonic anhydrase inhibitor acetazolamide, could significantly reduce AQP4 activity [84].

The carbonic anhydrase inhibitor acetazolamide and anti-epileptic drugs such as topiramate and zonisamide [85] have been reported to inhibit ectopically expressed AQP4 in *Xenopus* oocytes [84] as well as purified AQP4 reconstituted in liposomes [86]; however, other groups have been unable to confirm this activity using AQP4-transfected thyroid epithelial cells and primary glial cultures [87].

Bumetanide is known as a loop diuretic drug that inhibits the NKCC cotransporter in the ascending limb of the loop of Henle in the kidneys [88] and was selected as the structural scaffold for designing a chemical library of derivatives based on its small but significant blockade of AQP4 water channel function [89]. Bumetanide as well as its derivatives were shown to inhibit AQP4 in *Xenopus* oocytes.

As stated above, there are several reports of AQP4 inhibitors (including acetazolamide, anti-epileptic drugs, bumetanide, thiadiazole and others); unfortunately, follow-up studies of these compounds by other investigators using different assays have failed to confirm AQP4 inhibition. For a detailed discussion on the issues related to the discovery of AQP inhibitors, please refer to the recent review by Verkman et al. [90]. One interesting development is the identification of an autoantibody against AQP4 produced in patients with an inflammatory demyelinating disease of the CNS known as neuromyelitis optica; this antibody is referred to as AQP4-IgG or NMO-IgG [91]. Monoclonal AQP4-IgG has been successfully produced artificially [92], and linking AQP4-IgG to a toxin could be delivery system for destroying glioblastoma cells, most of which express high levels of AQP4. Thus, AQP4-IgG has the potential to become widely available and specifically engineered to kill AQP4-overexpressing cells in patients with malignant glioblastoma.

It is believed that identifying specific AQP4-selective inhibitors is currently a high priority in ongoing research. With an increasing number of novel compounds being tested, it is possible to begin elucidating a role of AQP blockers in mitigating pathologies such as brain oedema, glioblastoma, and breast and colon cancers as well as many other clinically important disorders [83]. Hopefully, improved chemotherapeutic targeting of AQP4 will be available to patients with malignant glioma in the near future.

## CONCLUSIONS

AQP4 is dissociated from the OAPs and redistributes across the entire surface of glioma cells under tumor conditions. AQP4 expression levels could correlate to the tumor grade, as AQP4 expression levels increase with higher glioma grades. More importantly, the redistribution of AQP4 and OAPs could be one of the earliest indicators of glioma transformation. However, the facilitating role of AQP4 in the infiltration and invasion of malignant glioblastoma cells requires additional research. Furthermore, the important roles of AQP4 in preventing drug resistance during glioma chemotherapy and of potential novel pharmacological blockers of AQP4 have been elucidated. We should be aware of the various unresolved questions regarding AQP4 in human glioblastoma. Further discovery of the dynamics of OAPs and AQP4 molecules in tumor may be critical to gain insight into the potential role of AQP4 in malignant glioma regarding the prevention, treatment, and classification of the tumor type and progression stages. Although the observations summarized in this review should be confirmed with more studies, we believe that they could provide critical information for the design of more focused research that will allow for systematic and definitive evaluation of the role of AQP4 in glioma treatments. More effort should be directed toward clarifying the newly discovered functions and molecular mechanisms of AQP4 in malignant gliomas.
